# Smartphone App–Based Eating Behavior Monitoring and Feedback Intervention for Glucocorticoid-Induced Appetite Increase in Patients With Systemic Lupus Erythematosus: Protocol for a Pilot Randomized Controlled Trial

**DOI:** 10.2196/78612

**Published:** 2025-12-15

**Authors:** Takashi Yamaguchi, Nobuyuki Takahashi, Ryohei Inanaga, Ryuhei So, Hiroe Kikuchi, Hisashi Noma, Hiroyuki Sasai, Kazuro Kamada, Tomohiro Sugimoto, Hiroshi Tsushima, Takanori Ichikawa, Hirofumi Miyake, Shunichi Fujita, Keisuke Ono, Yusuke Miwa, Anna Hasegawa, Naoki Suzuki, Akira Onishi, Toshihiro Matsui, Ryu Watanabe, Yasuhiro Hasegawa, Rei Ono, Takeo Isozaki, Yuichi Ishikawa, Nobuyuki Yajima, Noriaki Kurita

**Affiliations:** 1 Department of Hospital Pharmaceutics, School of Pharmacy, Showa Medical University, Shinagawa-ku, Japan; 2 Department of Nephrology, Shin-yurigaoka General Hospital, Kawasaki, Japan; 3 Department of Clinical Epidemiology, Graduate School of Medicine, Fukushima Medical University, 1 Hikarigaoka, Fukushima, 960-1295, Japan, 81 24-547-1470; 4 Department of Psychiatry, Okayama Psychiatric Medical Center, Okayama, Japan; 5 Department of Psychosomatic Medicine, National Center for Global Health and Medicine, Japan Institute for Health Security, Shinjuku-ku, Japan; 6 Department of Interdisciplinary Statistical Mathematics, The Institute of Statistical Mathematics, Tachikawa, Japan; 7 Research Team for Promoting Independence and Mental Health, Tokyo Metropolitan Institute for Geriatrics and Gerontology, Itabashi-ku, Japan; 8 Department of Rheumatology, Endocrinology and Nephrology, Faculty of Medicine and Graduate School of Medicine, Hokkaido University, Sapporo, Japan; 9 Department of Clinical Immunology and Rheumatology, Hiroshima University Hospital, Hiroshima, Japan; 10 Department of Internal Medicine and Rheumatology, Juntendo University Shizuoka Hospital, Izunokuni, Japan; 11 Department of Medicine (Neurology and Rheumatology), School of Medicine, Shinshu University, Matsumoto, Japan; 12 Department of General Internal Medicine, Tenri Hospital, Tenri, Japan; 13 Department of Rheumatology, Kawasaki Medical School, Kurashiki, Japan; 14 Department of Nephrology and Rheumatology, School of Medicine, Kyorin University, Mitaka, Japan; 15 Division of Rheumatology, Department of Medicine, Showa Medical University, Shinagawa-ku, Japan; 16 Department of Rheumatology, Dokkyo Medical University, Mibu, Japan; 17 Department of Stem Cell and Immune Regulation, Graduate School of Medicine, Yokohama City University, Yokohama, Japan; 18 Department of Advanced Medicine for Rheumatic Diseases, Kyoto University Graduate School of Medicine, Kyoto, Japan; 19 Department of Rheumatology Research, Clinical Research Center for Allergy and Rheumatology, National Hospital Organization Sagamihara National Hospital, Sagamihara, Japan; 20 Department of Clinical Immunology, Graduate School of Medicine, Osaka Metropolitan University, Osaka, Japan; 21 Department of Rheumatology, Kitasato University, Sagamihara, Japan; 22 Department of Pathogenesis and Translational Medicine, Graduate School of Pharmacy, Showa Medical University, Shinagawa-ku, Japan; 23 The First Department of Internal Medicine, University of Occupational and Environmental Health Japan, Kitakyushu, Japan; 24 Department of Innovative Research and Education for Clinicians and Trainees (DiRECT), Fukushima Medical University Hospital, Fukushima, Japan

**Keywords:** systemic lupus erythematosus, smartphone app, randomized controlled trial, eating behavior, appetite, glucocorticoid, ecological momentary assessment

## Abstract

**Background:**

Increased appetite and weight gain are common adverse effects of glucocorticoid therapy in patients with systemic lupus erythematosus (SLE). Concerns about appearance-related changes due to weight gain can reduce medication adherence. Moreover, the complex interplay among glucocorticoids, mood changes, sleep disturbances, and appetite can influence eating behaviors. Daily data collection using ecological momentary assessment and analysis of interrelations may help clarify these dynamics. Furthermore, real-time feedback based on daily eating behavior may help patients regulate appetite and eating patterns. Accordingly, we developed Mogu!☆Log, a smartphone-based app that enables daily self-reporting of eating behaviors, appetite, and mood and provides graphical feedback on meal frequency and perceived control over eating.

**Objective:**

This paper presents a protocol for a pilot randomized controlled trial designed to evaluate the effects of real-time feedback on eating behaviors using the Mogu!☆Log app among patients with newly diagnosed SLE who had started glucocorticoid therapy.

**Methods:**

This multicenter study recruited Japanese patients with newly diagnosed SLE who had started glucocorticoid therapy across 15 hospitals with rheumatology services. Participants were randomly assigned in a 1:1 ratio to two groups: (1) the immediate feedback group, which receives graphical feedback on meal frequency and perceived control over eating starting from day 1, and (2) the delayed feedback group, which uses the same app without feedback for the first 14 days and begins receiving identical feedback from day 15. Participants enter data daily from day 1 to day 21 after randomization. The primary outcome is the mean number of meals on day 14 after glucocorticoid initiation. Secondary outcomes include the loss-of-control-over-eating score and a 5-item visual analog scale–based appetite score, both recorded on day 14. Between-group mean differences will be analyzed using 2-tailed *t* tests. The target sample size is 60. In an embedded observational “study within a trial,” linear mixed models will examine whether glucocorticoid dose influences appetite scores through mood and sleep changes.

**Results:**

We hypothesized that participants receiving immediate feedback will have fewer meals on day 14, reduced loss of control over eating, and better appetite scores. The study received funding in April 2019, April 2022, and April 2024. Recruitment began in October 2024, and 17 participants had been enrolled as of May 2025. Data collection is expected to be completed by March 2027; data analysis has yet to begin. Results will be submitted for publication and reported to the University Hospital Medical Information Network (UMIN) registry in the summer of 2027.

**Conclusions:**

This pilot trial will provide foundational data on the feasibility and efficacy of smartphone-based real-time feedback in managing glucocorticoid-induced appetite increase in patients with SLE. These findings may contribute to the growing body of literature on app-based interventions for medication-related adverse effects.

## Introduction

### Clinical Relevance of Glucocorticoid-Related Side Effects in Systemic Lupus Erythematosus

Glucocorticoids are the cornerstone of initial treatment for systemic lupus erythematosus (SLE), a disease that predominantly affects young to middle-aged women [1]. However, prolonged glucocorticoid use is associated with serious medical complications, such as infections and osteoporosis, as well as adverse quality-of-life effects, including increased appetite, weight gain, insomnia, and mood changes [2-4]. More than 30% of glucocorticoid users report experiencing weight gain and insomnia [5]. Furthermore, online patient communities frequently describe appetite increases and weight gain as distressing experiences [1]. Despite their prevalence, these side effects are often underrecognized in clinical practice and underexplored in research, with limited practical guidance available in standard medical literature.

### Unclear Mechanisms Underlying Glucocorticoid-Induced Appetite Changes

Prolonged glucocorticoid therapy induces weight gain [1], and patients with Cushing disease—characterized by chronic endogenous glucocorticoid exposure—commonly report increased food cravings rather than hunger [6]. Although exogenous glucocorticoid administration has been shown to increase food intake [7], the specific components of appetite affected by glucocorticoids remain unclear, possibly due to the limited use of validated appetite assessment scales in previous studies. Furthermore, the complex interplay among glucocorticoid use, mood changes, sleep disturbance, and appetite changes is poorly understood. Clinical glucocorticoid use is known to alter mood (eg, depression and irritability) and cognitive function (eg, difficulty with concentration and memory) [8]. Negative emotional states, in turn, can trigger increased food intake in healthy populations [9,10]. Whether similar mechanisms underlie glucocorticoid-induced appetite changes in patients with SLE remains uncertain. Although glucocorticoid-related sleep disturbances, such as insomnia, increased percentage of time spent awake, and increased percentage of time spent in slow-wave sleep, are well documented [11], it remains unclear whether these disturbances lead to mood deterioration similar to that observed in healthy individuals or those with mood disorders [12,13].

### Potential of Ecological Momentary Assessment and Feedback Interventions

Ecological momentary assessment (EMA) enables real-time monitoring of behavior and symptoms, offering a promising approach to clarify the dynamic interrelationships among appetite, mood, and sleep. In addition, behavioral interventions that incorporate daily feedback may help patients manage their appetite and eating behaviors. Smartphone apps delivering real-time feedback have shown potential in promoting dietary self-regulation among adults with obesity [14].

By using brief questions with simple response options and automated, immediate data processing [15,16], these tools minimize user burden and enhance sustained engagement through real-time feedback [14]. Consequently, such approaches may also benefit patients with SLE who experience appetite increases and weight gain following glucocorticoid therapy.

However, evidence regarding the use of EMA with real-time feedback in patients with glucocorticoid-treated SLE is limited. Although a previous study used an EMA-based smartphone app to assess meal frequency in healthy adults, most studies lack validated appetite measures or control groups [17]. Furthermore, existing EMA studies involving patients with SLE have primarily focused on sleep quality, without assessing appetite or mood [18].

### Development of a Smartphone-Based Monitoring and Feedback Tool

To address these gaps, we developed Mogu!☆Log (もぐ！☆ログ), a smartphone app designed to monitor and provide feedback on appetite, mood, and eating behavior. The app allows daily self-reporting and visual feedback and includes a built-in randomization function to assess the impact of feedback on behavior. By integrating clinical and sleep data, this tool facilitates a comprehensive EMA-based analysis of how glucocorticoid therapy influences appetite through mood- and sleep-related pathways.

### Objectives

This study aims to (1) evaluate the effect of real-time dietary feedback on meal frequency in patients newly diagnosed with SLE who have started with glucocorticoid therapy and (2) examine how glucocorticoids affect appetite through mood and sleep changes. We will conduct a pilot randomized controlled trial (RCT) to evaluate the feedback intervention, along with an embedded observational cohort study to explore interrelationships using multilevel modeling. This protocol is reported in accordance with the Standard Protocol Items: Recommendations for Interventional Trials (SPIRIT) guidelines [19] (Checklist 1).

## Methods

### Study Setting

This study is conducted in the rheumatology departments of 15 general or university hospitals in Japan, each comprising both outpatient and inpatient services. These hospitals were selected based on their established collaborative research relationships with the principal investigators (NK and NY) and their experience managing newly diagnosed patients with SLE.

### Eligibility Criteria

#### Inclusion Criteria

Inclusion criteria include patients who (1) are diagnosed with SLE based on the 1997 American College of Rheumatology classification criteria [20] and (2) have initiated glucocorticoid therapy for the first time, with dosage determined at the discretion of the treating physician. There are no age restrictions; however, as rheumatology departments primarily serve adult populations, the inclusion of minors is expected to be rare. Although SLE predominantly affects women, male patients are also eligible, and sex is not used as an inclusion criterion. Participants must be able to understand Japanese because the mobile app and all study materials are available only in Japanese.

#### Exclusion Criteria

Exclusion criteria include patients who (1) have neuropsychiatric lupus with seizures, confusion, disorientation, or delusions or (2) are unable to access the Mogu!☆Log app via the LINE platform (LY Corp).

### Intervention

**Figure 1.**
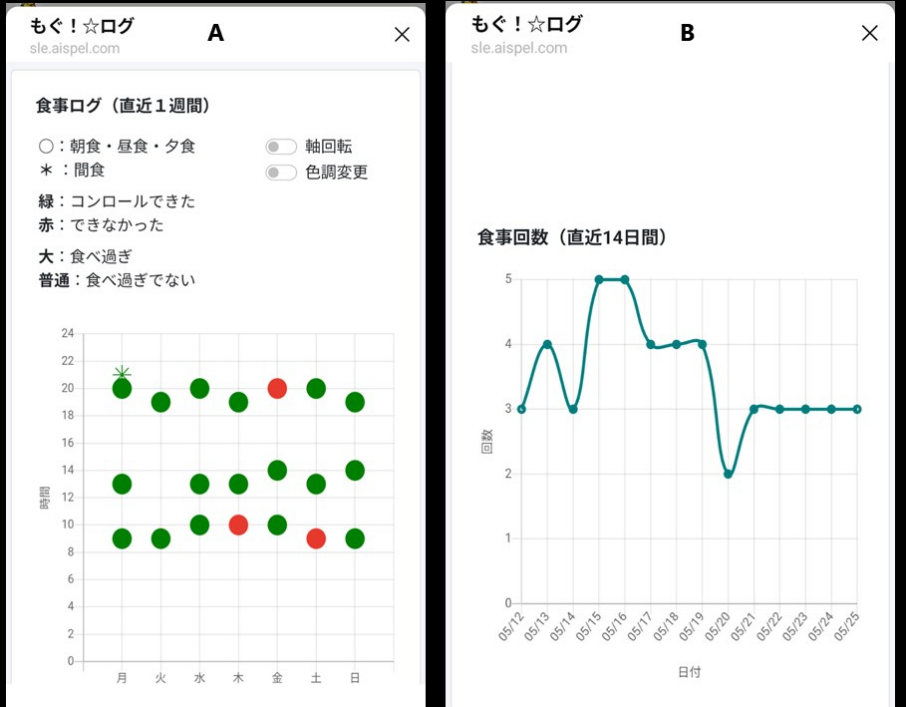
Feedback interface of the Mogu!☆Log smartphone app on the LINE social networking platform. (**A**) Graphical feedback showing self-evaluated eating episodes. Data from the past 7 days are plotted, with days on the x-axis and time (midnight to 11 PM) on the y-axis. Label size indicates perceived overeating (larger for episodes rated as overeating), whereas label color reflects perceived loss of control (red for loss of control, green otherwise). (**B**) Graphical feedback showing the frequency of eating episodes. Data from the past 14 days are presented, with dates on the x-axis and the number of episodes on the y-axis.

Participants use Mogu!☆Log, a smartphone-based app delivered via the LINE platform, to record meal frequency and episodes of loss of control over eating. The intervention group receives real-time graphical feedback immediately after each entry, displaying data from up to the previous 7 days (Figure 1; English translation of the Japanese text displayed in the app is provided in Multimedia Appendix 1).

Two types of feedback are provided. The first graph summarizes eating episodes over the past 7 days, indicating whether overeating or loss of control occurred (Figure 1A). The second graph presents daily meal counts over the past 14 days (Figure 1B).

**Figure 2.**
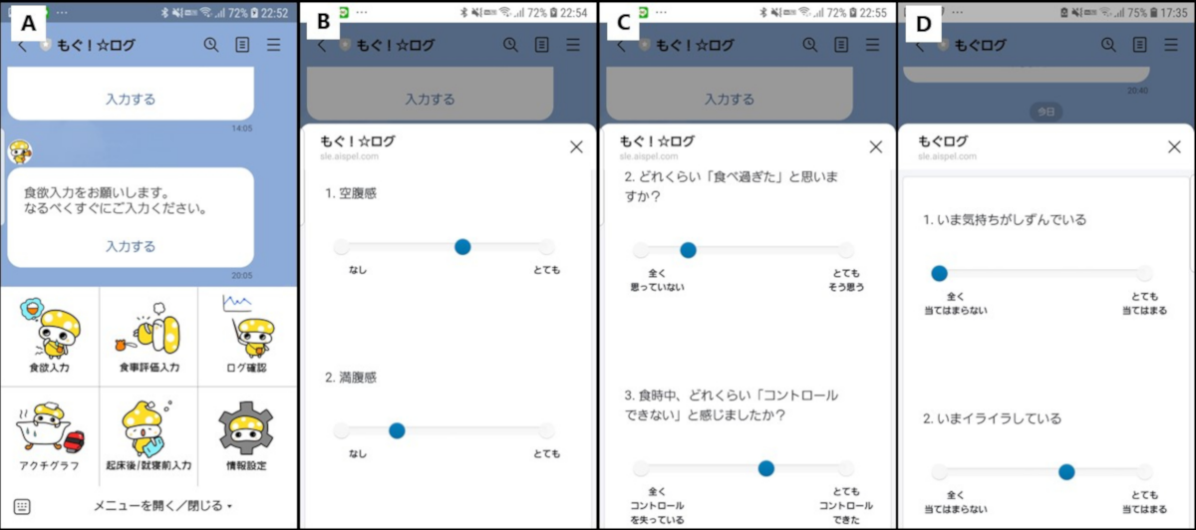
Widget interface of the Mogu!☆Log smartphone app on the LINE social networking platform. (A) Main menu widget: clockwise from the top left—Appetite, Eating Behavior Evaluation, Log Review, User Settings, Morning/Night Entry, and Actigraphy Attachment. (B) Visual analog scale (VAS) input for appetite: users slide bars to rate hunger and satiety. (**C**) VAS input for eating episodes: users rate the degree of perceived overeating and loss of control during meals. (**D**) VAS input for mood: users rate depressed mood and irritation. Values for VAS inputs are automatically calculated based on the ratio of a slider’s position (distance from the left) to the full length between labeled end points.

Participants register the day before glucocorticoid initiation and record data from treatment days 1 to 21 (Figure 2; English translation of the Japanese text displayed in the app is provided in Multimedia Appendix 1). Appetite and mood are assessed before and 2 hours after each meal. Overeating and loss of control are recorded immediately after every eating episode, including snacks. Before starting the study, participants received instructions on voluntarily logging entries before and after meals. The app automatically sends reminders for daily reporting. Reminders for postprandial entries (2 hours after a meal) were automatically scheduled based on each participant’s self-registered typical meal times (breakfast, lunch, and dinner). Specifically, these reminders were delivered randomly within +30 or −30 minutes of 2 hours after each registered mealtime (eg, if breakfast was set at 8 AM, the reminder was sent randomly between 9:30 and 10:30 AM). In addition, for immediate postmeal entries, if a participant did not submit an entry within 60 minutes after a premeal report, an automated reminder was sent.

The immediate feedback group receives graphical feedback from day 1, whereas the delayed feedback group does not receive feedback until day 14. After that, participants receive identical real-time feedback as the intervention group. The 14-day delay was determined for the following reasons: (1) at least 7 days of data are required to capture weekly behavioral patterns [15]; (2) a feedback duration longer than 1 week is desirable to detect behavioral change [17]; and (3) the accelerometer device used in the embedded “study within a trial” (SWAT) has a battery life of approximately 21 days, determining the total study duration. Accordingly, data collection spans 21 days, comprising a 14-day intervention or control phase followed by a 7-day delayed feedback phase, ensuring both groups eventually receive feedback exposure.

To encourage adherence, participants receive Amazon e-gift cards at registration and on days 7, 14, and 21 with the amounts corresponding to their logging activities.

### Outcome Measures

#### Primary Outcome

The primary outcome is the mean number of meals on day 14 after glucocorticoid initiation. This measure was selected to assess the impact of graphical feedback on eating behaviors. Meal frequency has previously been used as an index of the effectiveness of app-based feedback interventions [17].

Feasibility outcomes, including the completion rate and SD of the primary outcome, will also be reported to inform sample size estimation and refine the protocol for a future full-scale trial.

#### Secondary Outcomes

Secondary outcomes include loss of control before meals, loss of control during meals, and appetite, all assessed on day 14. Loss of control is measured using a visual analog scale (VAS) comprising 2 items: before meals, “To what extent do you feel in control of your eating?” (0‐100 scale); during meals, “During meals, how much did you feel out of control?” (0‐100 scale). Appetite is measured using a validated 5-item VAS-based scale for EMA [21].

We will also examine the 21-day trajectories of loss of control (ie, before and during meals) and appetite. Given the limited evidence on how glucocorticoid affects appetite over time, these trajectories will be analyzed separately for each feedback group.

#### Participant Timeline

Patients diagnosed with SLE and scheduled to begin glucocorticoid therapy are recruited 1 to 14 days before treatment initiation (Table 1). From days 1 to 21, all participants use the mobile app to report their mood, appetite, and eating behaviors. Participants also wear an actigraph continuously—except while bathing—to monitor their activity and sleep. Paper-based psychological and behavioral questionnaires are administered on days 1 and 21.

**Table 1.** Participant timeline.

Time point in study timeline	Enrollment (day −14 to day −1)	Randomization (day 0)	Day 1 after randomization	Day 14 after randomization	Day 15 after randomization	Closeout (day 21)
Enrollment						
Eligibility assessment	✓					
Informed consent	✓					
Randomization		✓				
Intervention						
Immediate feedback			✓	✓	✓	✓
Delayed feedback					✓	✓
Assessment						
Baseline variables^a^	✓					
EMA^b^-based mood, appetite, and eating behavior^c^			✓	✓	✓	✓
Medication^d^			✓	✓	✓	✓
Actigraph-based physical activity and sleep data^e^			✓	✓	✓	✓
Paper-based questionnaire^f^			✓			✓

a Participant characteristics, including sex, birth date, age, psychiatric/psychosomatic outpatient status, height, weight, and disease activity (systemic lupus erythematosus disease activity index 2000).

b EMA: ecological momentary assessment.

c Sleep parameters, wake-up time, bedtime, mood (before sleep and before and after meals), appetite (before meals), perceived control over eating (before and after meals), and subjective assessment of meal quantity (after meals).

d Including glucocorticoids (prednisone-equivalent dose per day), psychotropic medications (hypnotics, antiepileptics, mood stabilizers, and antidepressants), hydroxychloroquine, and immunosuppressants.

e Including activity levels, nocturnal awakenings, wake after sleep onset, sleep onset latency, and sleep efficiency.

f A Three-Factor Eating Questionnaire-R18V2, Athens Insomnia Scale, Health-Related Hope Scale, and Short Grit Scale.

### Sample Size

The target sample size is 60 participants, taking into account the rarity of newly diagnosed SLE and the constraints of study duration and funding. This pilot trial aims to obtain key data for planning a future trial, ensuring at least 30 participants to estimate parameters for outcome measures (particularly SDs), as recommended in the pilot study design literature [22].

### Recruitment

Principal investigators (NK and NY) recruited rheumatologists from 15 hospitals across 5 Japanese regions (Hokkaido, Kanto, Chubu, Kinki, and Chugoku) through professional networks. Despite the rarity of SLE, recruitment was deemed feasible in both outpatient and inpatient hospital settings.

### Assignment of Interventions

The procedures for randomization and blinding are as follows:

- Allocation—the app incorporates a simple randomization algorithm for participant assignment.
- Concealment—allocation is centrally randomized and concealed from both patients and physicians at enrollment.
- Implementation—physicians enroll patients, while the app performs group allocation automatically.
- Blinding—participants are blinded, although group inference may be possible based on feedback timing (health care providers remain blinded unless they view the participant’s screen; data analysts receive deidentified datasets without group assignment indicators).

### Data Collection and Management

Data were collected as follows: meal frequency was recorded electronically through user taps in the app at each eating occasion. VAS scores for loss of control, mood, and appetite were collected via in-app sliders with labeled anchors. Physical activity and sleep parameters, including activity levels, nocturnal awakenings, wake after sleep onset, sleep onset latency, and sleep efficiency, were measured using an actigraph (Table 1). These data were analyzed as outcomes or covariates in the SWAT.

Validated paper-based questionnaires included the Three-Factor Eating Questionnaire-R18V2 [23,24], Athens Insomnia Scale [25,26], Health-Related Hope Scale [27], and Short Grit Scale [28].

To promote retention, participants received Amazon e-gift cards on days 7, 14, and 21, with amounts proportional to the number of completed app entries.

Data management was conducted as follows: app data were securely transmitted and stored on an encrypted server. Input options were restricted to predefined ranges to ensure data validity. Paper-based data were double entered and cross-checked to identify discrepancies.

### Statistical Analysis

For the primary outcome, meal frequency on day 14 will be compared between groups using a 2-sided Student *t* test with a 5% significance level. For sensitivity analysis, an analysis of covariance will be performed, adjusting for disease activity and glucocorticoid dose.

For the secondary outcomes, between-group comparisons of loss-of-control and appetite scores on day 14 will be conducted using the 2-tailed Student *t* test. Sensitivity analyses with analysis of covariance adjusted for disease activity and glucocorticoid dose will be conducted. Longitudinal changes over the 21-day period will be analyzed using linear mixed-effects models with random intercepts for individual participants and fixed effects for group, time, and group-by-time interactions.

For the observational SWAT, linear mixed-effects models will be used to examine associations between glucocorticoid dose and outcomes such as appetite, snacking behavior, and loss of control. Mood and sleep variables will also be analyzed using the same modeling framework to assess the effect of glucocorticoid dosage. Additionally, linear mixed-effects models will be used to assess whether mood and sleep mediate the relationship between glucocorticoid dose and appetite. All models will incorporate random intercepts for individual participants and fixed effects for group, time, and group-by-time interactions.

### Monitoring, Harms, and Auditing

No formal monitoring is planned due to the noninvasive nature of the intervention and the pilot study design. No interim analyses, harm assessments, or audits will be conducted.

### Ethical Considerations

#### Ethics Approval and Consent

This study received centralized ethics approval from the Ethics Review Board of Fukushima Medical University (REC2023-097). Any protocol amendments, including changes in investigators or participating institutions, will be made as required. Eligible participants will receive an approved explanation sheet from collaborating investigators and provide written informed consent before enrollment. Participants will receive compensation in the form of Amazon e-gift cards corresponding to their logging activities, as detailed in the intervention section.

#### Confidentiality

App-entered data are securely transmitted and stored on a protected server. Paper-based data are mailed to the research office and digitized using double data entry to maintain confidentiality and data accuracy.

#### Access to Data

The principal investigator (NK) will have access to the final dataset. A deidentified and blinded version will be shared with the biostatistician (HN) for analysis of the pilot trial.

#### Dissemination Policy

Study results will be disseminated through peer-reviewed publications. Ongoing progress and updates will be reported regularly on the trial registry (UMIN000052113).

## Results

This study received funding in April 2019, April 2022, and April 2024. App development was completed in March 2022. The first ethics approval was obtained in September 2023. Participant recruitment began in October 2024. As of May 2025, a total of 17 participants have been enrolled. Data collection is scheduled for completion by March 2027, after which data analysis will be conducted. The study findings will be submitted to peer-reviewed journals and reported on the UMIN trial registry in summer 2027.

## Discussion

### Overview and Significance of the Study

This study evaluates the feasibility and preliminary effectiveness of real-time, app-based feedback for managing eating behaviors and self-regulation following glucocorticoid therapy. Conducted through a multicenter collaboration across 15 rheumatology centers, this study uses a hybrid design that combines a pilot RCT with an embedded observational SWAT.

The RCT aims to assess the effect of feedback delivered via the Mogu!☆Log smartphone app on meal frequency and perceived appetite control. In parallel, the SWAT uses EMA through the same app to explore the interrelationships among glucocorticoid-induced changes in appetite, mood, physical activity, and sleep.

### Advantages and Challenges of Real-Time Graphical Feedback

Advances in mobile health technologies have heightened interest in app-based self-monitoring and feedback interventions. For patients starting glucocorticoid therapy, monitoring appetite and eating behaviors may help mitigate glucocorticoid-induced hyperphagia. However, the burden of frequent data entry may reduce adherence, and interventions lacking validation should be applied with caution. A previous study in the general population reported that dietary logging apps reduced meal frequency and prolonged intervals between meals [17]; however, its single-arm, pretest-posttest design limited causal inference. To address this limitation, this study compares immediate and delayed (2-week) graphical feedback, allowing for direct evaluation of the specific impact of real-time feedback on eating behavior. Furthermore, examining changes in eating behavior between days 14 and 21 in the delayed feedback group—after feedback is introduced—may further clarify the behavioral impact of this intervention.

### Addressing Appetite Increase and Weight Gain in Glucocorticoid Therapy

Increased appetite and weight gain are among the most common and distressing adverse effects of glucocorticoid therapy in patients with SLE [2-4]. These side effects, specifically appearance-related concerns, negatively impact medication adherence [3,29]. Despite their clinical importance, discrepancies often exist between physicians and patients in how they perceive the significance of glucocorticoid-related adverse effects [30], underscoring the need for patient-centered interventions. This study specifically targets glucocorticoid-induced appetite increase and weight gain—symptoms frequently reported by patients as particularly burdensome. While a previous study in the general population links emotional distress and loss of appetite control to overeating [31], such findings may not be generalizable to individuals undergoing glucocorticoid therapy. This study aims to clarify how neuropsychiatric effects of glucocorticoids influence eating behavior and to inform the development of future smartphone-based interventions that address medication-related side effects.

In medical contexts, the neuropsychiatric effects of glucocorticoid therapy observed during its early stages typically involve mood alterations (eg, depression and irritability) and cognitive changes (eg, difficulty with concentration and memory) [8]. The mood dimension examined in this study primarily reflects valence—including displeasure—within the framework of Russell’s circumplex model of affect, which describes affect along the dimensions of valence and arousal [32]. Focusing on valence is reasonable because a study in a nonclinical population demonstrated that transient increases in endogenous glucocorticoid levels were associated with negative affect (low valence), whereas no association with arousal was observed [33].

### Importance of an Interdisciplinary Collaborative Framework

This study integrates high-frequency, real-time EMA via a smartphone platform within an interdisciplinary research framework. Experts in rheumatology, EMA, eating behavior, and psychiatry collaborated to ensure appropriate sampling schedules and select validated measurement instruments aligned with the study objectives. Capturing dynamic behavioral changes during glucocorticoid therapy necessitates a research design that balances methodological rigor with participant burden. This interdisciplinary approach strengthens both the scientific quality and clinical relevance of the study, thereby increasing its potential for real-world application.

### Limitations and Future Directions

This study has some limitations. First, the small sample size inherent to a pilot study design limits both statistical power and generalizability. Larger-scale trials will be needed to validate these preliminary findings. Second, the requirement to use the LINE app may introduce selection bias by excluding individuals without compatible smartphones or familiarity with the app. However, given that smartphone ownership among Japanese adults in the typical age range for SLE onset (aged 20 to 40 years) is 97% to 100% and LINE use is 84% to 92% [34], this bias is likely minimal. Third, although several app-based measures, such as those for mood and appetite control, were developed with interdisciplinary input to ensure content validity in the context of glucocorticoid therapy, their psychometric properties remain to be further evaluated. Additional validation studies will be necessary to establish their reliability and broader applicability.

## Supplementary material

10.2196/78612 Multimedia Appendix 1 English translation of the Japanese text displayed in the app.

10.2196/78612 Multimedia Appendix 2 Prompt used for improving the clarity and grammatical accuracy of the English text.

10.2196/78612 Checklist 1 SPIRIT 2025 checklist of items to address in a randomized trial protocol.

## Abbreviations

EMA: ecological momentary assessment
RCT: randomized controlled trial
SLE: systemic lupus erythematosus
SPIRIT: Standard Protocol Items: Recommendations for Interventional Trials
SWAT: study within a trial
VAS: visual analog scale
