# Correction: Smartphone App–Based Eating Behavior Monitoring and Feedback Intervention for Glucocorticoid-Induced Appetite Increase in Patients With Systemic Lupus Erythematosus: Protocol for a Pilot Randomized Controlled Trial

**DOI:** 10.2196/90010

**Published:** 2026-01-21

**Authors:** Takashi Yamaguchi, Nobuyuki Takahashi, Ryohei Inanaga, Ryuhei So, Hiroe Kikuchi, Hisashi Noma, Hiroyuki Sasai, Kazuro Kamada, Tomohiro Sugimoto, Hiroshi Tsushima, Takanori Ichikawa, Hirofumi Miyake, Shunichi Fujita, Keisuke Ono, Yusuke Miwa, Anna Hasegawa, Naoki Suzuki, Akira Onishi, Toshihiro Matsui, Ryu Watanabe, Yasuhiro Hasegawa, Rei Ono, Takeo Isozaki, Yuichi Ishikawa, Nobuyuki Yajima, Noriaki Kurita

**Affiliations:** 1 Department of Hospital Pharmaceutics, School of Pharmacy, Showa Medical University, Shinagawa-ku, Japan; 2 Department of Nephrology, Shin-yurigaoka General Hospital, Kawasaki, Japan; 3 Department of Clinical Epidemiology, Graduate School of Medicine, Fukushima Medical University, 1 Hikarigaoka, Fukushima, 960-1295, Japan, 81 24-547-1470; 4 Department of Psychiatry, Okayama Psychiatric Medical Center, Okayama, Japan; 5 Department of Psychosomatic Medicine, National Center for Global Health and Medicine, Japan Institute for Health Security, Shinjuku-ku, Japan; 6 Department of Interdisciplinary Statistical Mathematics, The Institute of Statistical Mathematics, Tachikawa, Japan; 7 Research Team for Promoting Independence and Mental Health, Tokyo Metropolitan Institute for Geriatrics and Gerontology, Itabashi-ku, Japan; 8 Department of Rheumatology, Endocrinology and Nephrology, Faculty of Medicine and Graduate School of Medicine, Hokkaido University, Sapporo, Japan; 9 Department of Clinical Immunology and Rheumatology, Hiroshima University Hospital, Hiroshima, Japan; 10 Department of Internal Medicine and Rheumatology, Juntendo University Shizuoka Hospital, Izunokuni, Japan; 11 Department of Medicine (Neurology and Rheumatology), School of Medicine, Shinshu University, Matsumoto, Japan; 12 Department of General Internal Medicine, Tenri Hospital, Tenri, Japan; 13 Department of Rheumatology, Kawasaki Medical School, Kurashiki, Japan; 14 Department of Nephrology and Rheumatology, School of Medicine, Kyorin University, Mitaka, Japan; 15 Division of Rheumatology, Department of Medicine, Showa Medical University, Shinagawa-ku, Japan; 16 Department of Rheumatology, Dokkyo Medical University, Mibu, Japan; 17 Department of Stem Cell and Immune Regulation, Graduate School of Medicine, Yokohama City University, Yokohama, Japan; 18 Department of Advanced Medicine for Rheumatic Diseases, Kyoto University Graduate School of Medicine, Kyoto, Japan; 19 Department of Rheumatology Research, Clinical Research Center for Allergy and Rheumatology, National Hospital Organization Sagamihara National Hospital, Sagamihara, Japan; 20 Department of Clinical Immunology, Graduate School of Medicine, Osaka Metropolitan University, Osaka, Japan; 21 Department of Rheumatology, Kitasato University, Sagamihara, Japan; 22 Department of Pathogenesis and Translational Medicine, Graduate School of Pharmacy, Showa Medical University, Shinagawa-ku, Japan; 23 The First Department of Internal Medicine, University of Occupational and Environmental Health Japan, Kitakyushu, Japan; 24 Department of Innovative Research and Education for Clinicians and Trainees (DiRECT), Fukushima Medical University Hospital, Fukushima, Japan

In “Smartphone App–Based Eating Behavior Monitoring and Feedback Intervention for Glucocorticoid-Induced Appetite Increase in Patients With Systemic Lupus Erythematosus: Protocol for a Pilot Randomized Controlled Trial” [1], the authors noted an error.

The eighth author’s name has been changed from the following:

Kazuhiro Kamada

The name now appears as:

Kazuro Kamada

The correction will appear in the online version of the paper on the JMIR Publications website, together with the publication of this correction notice. Because this was made after submission to PubMed, PubMed Central, and other full-text repositories, the corrected article has also been resubmitted to those repositories.
