# Supplementary figures and images for: Smartphone App–Based Eating Behavior Monitoring and Feedback Intervention for Glucocorticoid-Induced Appetite Increase in Patients With Systemic Lupus Erythematosus: Protocol for a Pilot Randomized Controlled Trial
Source: JMIR Res Protoc. 2025 Dec 15;14:e78612. doi: 10.2196/78612 (PMC12705127; doi:10.2196/78612)

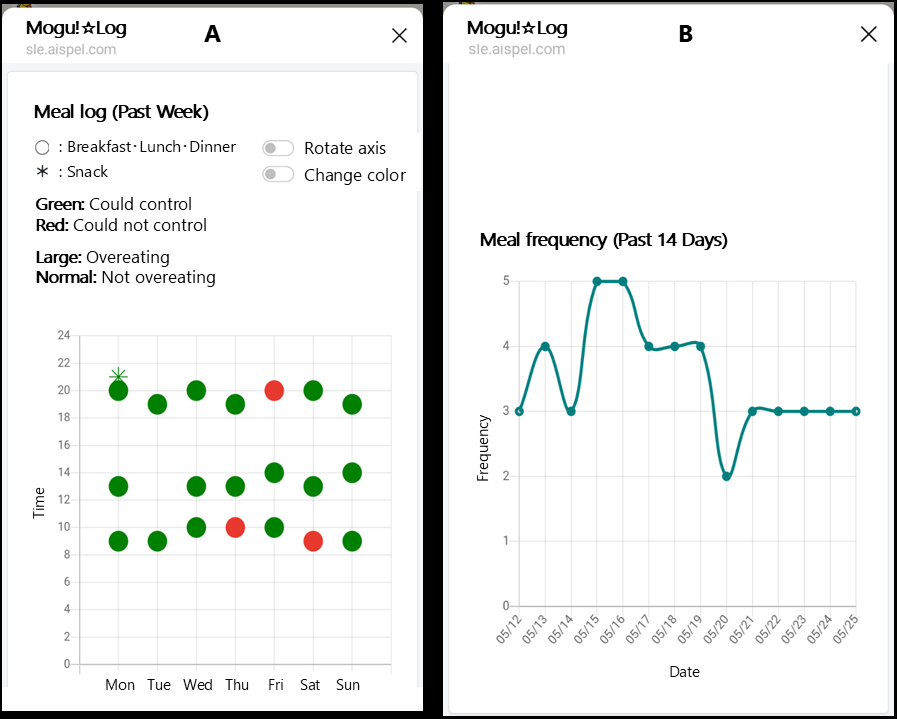

Supplement: Multimedia Appendix 1 [file resprot-v14-e78612-s001.png]
