# Supplementary material for: Smartphone App–Based Eating Behavior Monitoring and Feedback Intervention for Glucocorticoid-Induced Appetite Increase in Patients With Systemic Lupus Erythematosus: Protocol for a Pilot Randomized Controlled Trial
Source: JMIR Res Protoc. 2025 Dec 15;14:e78612. doi: 10.2196/78612 (PMC12705127; doi:10.2196/78612)
Supplement: Multimedia Appendix 2 [file resprot-v14-e78612-s002.docx]

Multimedia Appendix 3. Prompt used for improving the clarity and grammatical accuracy of the English text.

The authors used the following prompt when utilizing ChatGPT: Please edit these sentences written for an academic journal as if you were a native English speaker.
